# Residency programs and the outlook for occupational and environmental medicine in Korea

**DOI:** 10.1186/s40557-015-0072-1

**Published:** 2015-09-25

**Authors:** Youngil Lee, Jungwon Kim, Yoomi Chae

**Affiliations:** Department of Occupational and Environmental Medicine, Kosin University Hospital, 34-1 Amnam-dong, Seo-gu, Busan 602-702 Korea; Department of Occupational and Environmental Medicine, College of Medicine, Kosin University, 34-1 Amnam-dong, Seo-gu, Busan 602-702 Korea; Department of Occupational and Environmental Medicine, College of Medicine, Dankook University, san 29, Anseo-dong, Dongnam-gu, Cheonan-si, Chungnam 330-714 Korea

**Keywords:** Residency programs, Outlook, Occupational and environmental medicine

## Abstract

**Objectives:**

This study investigated the implementation of training courses and the overall outlook for occupational and environmental medicine (OEM) in Korea. We described the problems facing OEM residency programs in Korea, and reviewed studies dealing with the specialty of occupational health in developed countries in order to suggest directions of improvement for the OEM training courses.

**Methods:**

We surveyed 125 OEM residents using a questionnaire in August 2012. A total of 23 questions about the training environment, residency programs, preferred institutions for post-licensure employment, and the outlook for OEM specialists were included in the questionnaire and analyzed according to the type of training institution and residency year. Responses from 88 residents (70.4 %) were analyzed.

**Results:**

The major responsibilities of OEM residents were found to vary depending on whether they were trained in research institutes or in hospitals. OEM residents had a lower level of satisfaction with the following training programs: toxicology practice (measurements of biological markers, metabolites, and working environments), and OEM practice (environmental diseases and clinical training involving surgery). When asked about their eventual place of employment, OEM residents preferred institutions providing special health examinations or health management services. OEM residents reported a positive outlook for OEM over the next 5 years, but a negative outlook for the next 10 years.

**Conclusions:**

Although a standardized training curriculum for OEM residents exists, this study found differences in the actual training courses depending on the training institution. We plan to standardize OEM training by holding a regional conference and introducing open training methods, such as an open hospital system. Use of Korean-language OEM textbook may also reduce differences in the educational programs of each training institution. Toxicology practice, environmental diseases, and clinical training in surgery are areas that particularly need improvement in OEM residency training programs.

## Introduction

The field of occupational and environmental medicine (OEM) is particularly broad, ranging from clinical single patient-oriented medicine to public health [1]. OEM has dramatically changed during the past 20 to 30 years in response to new trends in both occupational diseases and employment [2]. Since the introduction of OEM specialization in 1996, the Korean Society of OEM has trained 666 OEM specialists as of 2014 [3, 4]. The medical practice of OEM is continually evolving in response to the increased participation of non-regular workers and women in the workforce, the aging of the workforce, the shift from manufacturing to a service-based economy, the appearance of new toxic substances such as nanoparticles, and the shift from traditional occupational diseases to work-related disorders. For these reasons, OEM competencies must be updated, and corresponding changes must be made to OEM residency programs. Although OEM residency training has been studied in other countries [1, 2, 5–7], few such studies have been performed in Korea [8]. Analogously, residency programs in other medical specialties have been better studied [9–16].

With the introduction of special health examinations for night shift workers, 1.3 million workers are expected to undergo special health examinations relating to night shift work annually, in 2016. [17]. Furthermore, in response to increasing concerns about the potential risks and hazards of exposure to chemicals and radiation, the number of hospitals and institutes that require OEM physicians is increasing. The Korean Industrial Safety and Health Act mandates that employers conduct periodic occupational medical examinations and provide health management services. The current tasks of most OEM physicians are special health examinations and health management services, and their main responsibilities are therefore sensitive to policy changes. Some institutions have requested that laws concerning the qualifications of doctors who perform special health examinations and health management services be revised [3, 18], which may lead to instability in the employment outlook for OEM physicians.

This study describes directions for future improvement by evaluating the implementation of current training courses for OEM residents. In addition, our survey inquired about the actual training courses, the satisfaction of residents with OEM residency programs, preferred institutions for employment after acquiring licensure as an OEM specialist, and the outlook for OEM specialists.

## Materials and methods

### Study subjects

The survey was conducted over the course of 3 months from August to October 2012. We distributed the questionnaires during a resident training lecture on August 25, 2012 to 125 OEM residents working in 32 OEM training institutions. A total of 92 questionnaires were returned, yielding a response rate of 73.6 %. In the final analysis, 88 (70.4 %) questionnaires were used, since some responses were discarded due to the absence of answers for more than 30 % of the survey questions.

### Study methods

The survey consisted of 23 questions asking about the training environment, residency programs, preferred institutions for post-licensure employment, and the perceived outlook for OEM specialists. The responses included no personal identifiers, and all information was handled confidentially. The responses were analyzed according to the type of training institution and resident year. In particular, satisfaction with various OEM training curricula, preferred institutions for employment after acquiring licensure as an OEM specialist, and the perceived outlook for OEM specialists were assessed using a six-point Likert scale, based on which the mean and standard deviation of the results were calculated. For satisfaction with various OEM training curricula, scores were given as follows: one point for ‘not satisfied at all,’ two points for ‘not satisfied,’ three points for ‘slightly dissatisfied,’ four points for ‘slightly satisfied,’ five points for ‘satisfied,’ and six points for ‘very satisfied’. A six-point Likert scale was used to indicate preferences for each type of post-licensure employment institution, with 1 point being ‘very negative’ and six points being ‘very positive.’ A six-point Likert scale was also used to assess the perceived outlook for OEM specialists, with 1 point representing ‘very negative’ and six points representing ‘very positive’ outlooks. The OEM residents were divided into those training at a research institute and those training at a hospital. Responses were compared between these two groups. OEM residents were also categorized into two groups according to their year in residency (1 to 2 years in training versus 3 to 4 years in training), in order to see if this criterion affected the responses.

The institute training group (ITG) consisted of residents training at the College of Medicine at Seoul National University, the Graduate School of Public Health at Seoul National University, the Graduate School of Public Health at Yonsei University, the College of Medicine at Ewha Womans University, and the Occupational Safety and Health Research Institute (OSHRI). The hospital training group (HTG) consisted of residents training in hospitals, including university hospitals. The lower-year group (LYG) was defined as residents in their first or second year of training and the higher-year group (HYG) was defined as residents in their third or fourth year of training.

For statistical analysis, continuous variables were evaluated using the Mann-Whitney test or the Student’s *t*-test, while categorical variables were evaluated using the Fisher's exact test and the chi-squared test. All analyses were two-tailed, and *p*-values < 0.05 were considered to indicate statistical significance. All statistical tests were performed using SPSS version 21.0 (IBM Corp., Armonk, NY, USA).

## Results

### General characteristics of OEM residents and training environments

Table 1 summarizes the general characteristics of the 125 OEM residents surveyed in August 2012. Of the 88 OEM residents who replied to the survey, nine (10.2 %) were in the ITG and 79 (89.8 %) were in the HTG. A total of 46 residents (52.3 %) were in the LYG and 42 (47.7 %) were in the HYG. Excluding clinical training in other departments, the distribution of responsibilities for the 88 OEM residents was as follows: practical training (51.7 % of their time), thesis/research-related work (30.6 % of their time), and other work (17.7 % of their time). When respondents were subdivided into the ITG and the HTG, the proportions of each responsibility were as follows: practical training (18.0 % vs. 56.1 % of their time, respectively), thesis/research-related work (58.5 % vs. 26.9 % of their time, respectively), and other work (23.5 % vs. 17.0 % of their time, respectively). Statistically significant differences between the ITG and the HTG were found in the proportions of each responsibility, the extent of off-site work, the major responsibilities involved in off-site work, and clinical training. Also, there was a statistically significant difference between the LYG and the HYG for the extent of off-site work, the major responsibilities involved in off-site work, and clinical training (Table 2).

**Table 1:** General characteristics of the OEM residents surveyed in August 2012

Variables		Institution			Residency year		
	Total	Institute	Hospital	*P*-value ^*^	Lower	Higher	*P*-value ^*^
Gender ^a^							
Male	103 (82.4)	15 (78.9)	88 (83.0)	0.668	53 (84.1)	50 (80.6)	0.609
Female	22 (17.6)	4 (21.1)	18 (17.0)		10 (15.9)	12 (19.4)	
Age ^b^	31.7 ± 3.9	31.8 ± 3.7	31.6 ± 4.0	0.839	30.8 ± 3.8	32.5 ± 3.9	0.013
Working region ^a^							
Seoul metropolitan area	66 (52.8)	19 (100.0)	47 (44.3)	0.000	35 (55.6)	31 (50.0)	0.534
Other areas	59 (47.2)	0 (0.0)	59 (55.7)		28 (44.4)	31 (50.0)	

*OEM* Occupational and environmental medicine; *SD* Standard deviation

^*^Chi-squared test or Student’s *t*-test

^a^ N (%)

^b^ Mean ± SD

**Table 2:** General characteristics and working conditions of OEM residents according to training institution and residency year

Variables		Institution			Residency year		
	Total ^a^	Institute	Hospital	*P*-value ^*^	Lower	Higher	*P*-value ^**^
Gender ^b^							
Male	70 (79.5)	5 (55.6)	65 (82.3)	0.060	35 (76.1)	35 (83.3)	0.400
Female	18 (20.5)	4 (44.4)	14 (17.7)		11 (23.9)	7 (16.7)	
Age ^c^	31.7 ± 3.6	31.6 ± 2.8	31.8 ± 3.7	0.847	31.2 ± 3.8	32.4 ± 3.2	0.119
Working region ^b^							
Seoul metropolitan area	45 (51.1)	9 (100.0)	35 (44.9)	0.001	27 (58.7)	18 (42.9)	0.138
Other areas	43 (48.9)	0 (0.0)	43 (55.1)		19 (41.3)	24 (57.1)	
Other Specialty before OEM? ^b^							
Yes: practiced other specialty first	2 (2.4)	0 (0.0)	2 (2.6)	1.000	0 (0.0)	2 (5.0)	0.218
No: directly into OEM	83 (97.6)	9 (100.0)	74 (97.4)		45 (100.0)	38 (95.0)	
Working time (hours/day)^c^	9.7 ± 1.8	9.6 ± 1.5	9.7 ± 1.8	0.968	9.5 ± 1.4	10.0 ± 2.1	0.176
Sleeping time (hours/day)^c^	6.6 ± 1.1	6.8 ± 1.3	6.5 ± 1.1	0.735	6.6 ± 1.1	6.5 ± 1.1	0.678
Off-site work (days/week) ^c^	3.0 ± 1.5	2.1 ± 1.2	3.0 ± 1.5	0.066	2.4 ± 1.2	3.4 ± 1.5	0.001
Major responsibility when working outside the institution ^b^							
Health examinations	47 (63.5)	0 (0.0)	47 (74.2)	0.000	25 (69.4)	22 (57.9)	0.029
Health management services	10 (13.5)	0 (0.0)	10 (15.2)		1 (2.8)	9 (23.7)	
Research or epidemiological surveys	17 (23.0)	8 (100.0)	9 (13.6)		10 (27.8)	7 (18.4)	
Did you undergo clinical training? ^b^							
Yes	67 (77.9)	5 (55.6)	62 (80.5)	0.088	29 (64.4)	38 (92.7)	0.002
No	19 (22.1)	4 (44.4)	15 (19.5)		16 (35.6)	3 (7.3)	

*OEM* Occupational and environmental medicine; *SD* Standard deviation

^*^Fisher’s exact test or Mann-Whitney test

^**^Chi-squared test or Student’s *t*-test

^a^Due to missing values, the total number may be different for each variable

^b^N (%)

^c^Mean ± SD

### Satisfaction with OEM residency programs

The satisfaction levels associated with training curricula on work-relatedness evaluations, fitness for work evaluations, OEM theory, and special health examinations were 3.5 points or higher on average, with the majority of responses being positive. The scores for toxicology practice, environmental diseases, and clinical training in surgery were lower than 3.5 points, with the majority of responses being negative. The mean satisfaction with OEM theory training was 4.9 points in the ITG and 3.5 points in the HTG, which was found to be a statistically significant difference. Similarly, the mean satisfaction with OEM practice (environmental diseases) was 4.0 points in the ITG and 2.9 points in the HTG, which was a statistically significant difference (Table 3).

**Table 3:** Satisfaction with OEM residency programs

Training curricula		Training institution ^a^			Residency year ^a^		
	Total ^b^	Institute	Hospital	*P*-value ^*^	Lower	Higher	*P*-value ^**^
OEM theory ^c^	3.7 ± 1.3	4.9 ± 0.3 (9, 10.6)	3.5 ± 1.3 (76, 89.4)	0.000	3.8 ± 1.3 (45, 52.9)	3.5 ± 1.2 (40, 47.1)	0.248
OEM practice							
Clinical training (medical areas)	3.4 ± 1.3	2.5 ± 2.3 (6, 8.8)	3.5 ± 1.2 (62, 91.2)	0.334	3.1 ± 1.6 (28, 41.2)	3.6 ± 1.1 (40, 58.8)	0.194
Clinical training (surgical areas)	3.1 ± 1.3	2.5 ± 2.3 (6, 9.4)	3.1 ± 1.2 (58, 90.6)	0.551	2.7 ± 1.6 (25, 39.1)	3.6 ± 1.1 (39, 60.9)	0.122
Special health examinations	3.6 ± 1.2	2.4 ± 2.3 (5, 7.2)	3.7 ± 1.0 (64, 92.8)	0.281	3.4 ± 1.2 (30, 43.5)	3.7 ± 1.1 (39, 56.5)	0.271
Health management services	3.4 ± 1.3	3.2 ± 1.9 (5, 9.6)	3.5 ± 1.3 (47, 90.4)	0.952	3.1 ± 1.2 (17, 32.7)	3.6 ± 1.2 (35, 67.3)	0.155
Work-relatedness evaluations	3.8 ± 1.3	3.8 ± 1.7 (8, 11.8)	3.9 ± 1.2 (60, 88.2)	0.730	3.8 ± 1.5 (30, 44.1)	3.8 ± 1.1 (38, 55.9)	0.977
Fitness for work evaluations	3.8 ± 1.3	3.4 ± 1.7 (7, 11.5)	3.8 ± 1.3 (54, 88.5)	0.731	3.7 ± 1.6 (24, 39.3)	3.8 ± 1.1 (37, 60.7)	0.679
Environmental diseases	3.0 ± 1.4	4.0 ± 0.7 (5, 8.6)	2.9 ± 1.4 (53, 91.4)	0.047	3.4 ± 1.4 (24, 41.4)	2.6 ± 1.2 (34, 58.6)	0.026
Toxicology practice							
Working environment measurements	3.0 ± 1.5	3.7 ± 2.0 (6, 11.3)	2.9 ± 1.4 (47, 88.7)	0.144	3.2 ± 1.9 (18, 34.0)	2.9 ± 1.2 (35, 66.0)	0.538
Biologic markers, metabolite measurements	2.8 ± 1.5	3.7 ± 2.0 (6, 12.0)	2.8 ± 1.5 (46, 88.0)	0.164	3.2 ± 2.0 (16, 32.0)	2.7 ± 1.3 (34, 68.0)	0.361

*OEM* Occupational and environmental medicine; *SD* Standard deviation

^*^Mann-Whitney test

^**^Student’s *t*-test

^a^Mean ± SD (N, %)

^b^Due to missing values, the total number may be different for each variable

^c^Occupational diseases, toxicology, epidemiology

### Areas of employment after acquiring licensure as an OEM specialist

A total of 28 residents (31.8 %) indicated that hospitals providing special health examinations or health management services were their preferred choice of employment, followed by university hospitals or universities (*n* = 18, 21.4 %), the Korean Industrial Health Association (KIHA) or the Korea Medical Institute (KMI) (*n* = 12, 14.3 %), the position of a health manager in a major company (*n* = 10, 11.9 %), and others (*n* = 13, 16.0 %). When asked to describe the reasons for their choice, 46.8 % said ‘they think the compensation would be good’ or indicated ‘work environment and quality of life.’ The employment type that was the least preferred was opening one’s own clinic, a response given by 22 residents (27.2 %), followed by KIHA/KMI (*n* = 20, 24.7 %), a university hospital or university (*n* = 15, 18.5 %), the Ministry of Labor or the Ministry of the Environment (*n* = 12, 14.8 %), and others (*n* = 12, 14.8 %).

With regards to employment institution preferences, on average respondents rated the following types of institutions with an average of 3.5 points or higher, indicating mostly positive responses: hospitals that provide special health examinations or health management services, KIHA/KMI, the position of a health manager in a major company, and university hospitals or universities. The scores for worker health centers, public institutions (e.g., OSHRI), the Ministry of Labor or the Ministry of the Environment, and opening one’s own clinic were on average lower than 3.5 points, with the majority of responses being negative. The ITG rated their preference for public institutions (e.g., OSHRI) at 3.9 points, compared to 3.0 points in the HTG, which was a statistically significant difference. The ITG rated their preference for the Ministry of Labor or Ministry of the Environment at 3.8 points, compared to 3.0 points in the HTG, which was likewise a statistically significant difference (Table 4).

**Table 4:** Preferred institution for employment after acquiring licensure as an OEM specialist

	Training institution ^a^				Residency year ^a^		
Areas of employment	Total (*n* = 86)	Institute (*n* = 9)	Hospital (*n* = 77)	*P*-value ^*^	Lower (*n* = 44)	Higher (*n* = 42)	*P*-value ^**^
Korean Industrial Health Association or Korea Medical Institute	3.8 ± 1.1	3.7 ± 0.9	3.8 ± 1.3	0.633	4.0 ± 0.9	3.6 ± 1.4	0.131
Hospital (providing special health examinations or health management services)	4.1 ± 1.0	3.7 ± 1.0	4.1 ± 1.0	0.183	4.0 ± 1.0	4.2 ± 1.0	0.139
University hospital or university	3.6 ± 1.4	4.1 ± 0.6	3.5 ± 1.4	0.280	3.6 ± 1.3	3.6 ± 1.5	0.974
Public institutions (e.g., OSHRI)	3.1 ± 1.2	3.9 ± 0.7	3.0 ± 1.2	0.011	3.2 ± 1.1	3.0 ± 1.2	0.407
Ministry of Employment and Labor or Ministry of the Environment	3.1 ± 1.3	3.8 ± 0.9	3.0 ± 1.3	0.042	3.1 ± 1.1	2.8 ± 1.3	0.073
Major company (health manager)	3.7 ± 1.1	3.9 ± 0.8	3.7 ± 1.1	0.662	3.7 ± 1.0	3.7 ± 1.2	0.971
Private clinic	3.0 ± 1.1	2.7 ± 0.7	3.1 ± 1.2	0.375	3.0 ± 1.2	3.1 ± 1.1	0.571
Worker health center	3.2 ± 1.2	3.4 ± 1.1	3.2 ± 1.2	0.492	3.3 ± 1.0	3.1 ± 1.3	0.222

*OEM* Occupational and environmental medicine; *OSHRI* Occupational Safety and Health Research Institute; *SD* Standard deviation

^*^By Mann-Whitney test

^**^By Student’s *t*-test

^a^Mean ± SD

### Outlook for occupational and environmental specialists

The perceived outlook for OEM specialists in the next 5 years was on average higher than 3.5 points, indicating a mostly positive response, for the following areas: job satisfaction, employment, overall outlook, and possibilities for development. The perceived outlook regarding social position was lower than 3.5 points, and most responses were negative. Although the score for expected pay over the next 5 years was less than 3.5 points, when the responses regarding the perceived outlook for pay were classified as positive and negative, it was found that most responses were positive (57.5 % positive, 42.5 % negative). The outlook for OEM specialists over the next 10 years was on average greater than 3.5 points, with mostly positive responses, particularly for job satisfaction. However, the overall outlook and the outlook for social position, employment, and pay were assessed at less than 3.5 points on average, indicating mostly negative responses (Table 5). Although the score for possibilities for development over the next 10 years was less than 3.5 points, more responses were positive than negative (55.2 % positive, 44.8 % negative).

**Table 5:** Perceived outlook for OEM specialists in Korea

	Training institution ^a^				Residency year ^a^		
Category	Total (*n* = 87)	Institute (*n* = 9)	Hospital (*n* = 78)	*P*-value ^*^	Lower (*n* = 46)	Higher (*n* = 41)	*P*-value ^**^
Overall outlook							
Next 5 years	3.6 ± 1.1	4.1 ± 1.1	3.5 ± 1.1	0.095	3.8 ± 1.2	3.4 ± 1.1	0.075
Next 10 years	3.3 ± 1.2	3.9 ± 0.9	3.2 ± 1.2	0.069	3.6 ± 1.2	3.1 ± 1.1	0.045
Pay							
Next 5 years	3.4 ± 1.1	3.8 ± 1.0	3.4 ± 1.1	0.154	3.6 ± 1.1	3.3 ± 1.0	0.191
Next 10 years	3.0 ± 1.1	3.5 ± 0.9	2.9 ± 1.1	0.065	3.2 ± 1.1	2.7 ± 1.1	0.040
Social position							
Next 5 years	3.2 ± 1.1	4.1 ± 0.9	3.1 ± 1.1	0.004	3.4 ± 1.1	3.0 ± 1.0	0.096
Next 10 years	3.1 ± 1.2	4.2 ± 1.0	3.0 ± 1.1	0.002	3.3 ± 1.2	2.9 ± 1.1	0.093
Development possibility							
Next 5 years	3.6 ± 1.1	4.1 ± 0.7	3.5 ± 1.1	0.085	3.7 ± 1.2	3.4 ± 0.9	0.131
Next 10 years	3.4 ± 1.2	4.1 ± 0.7	3.3 ± 1.2	0.040	3.6 ± 1.2	3.2 ± 1.2	0.209
Employment							
Next 5 years	3.6 ± 1.1	4.2 ± 0.9	3.5 ± 1.1	0.025	3.7 ± 1.2	3.4 ± 0.9	0.192
Next 10 years	3.1 ± 1.2	3.8 ± 0.9	3.0 ± 1.1	0.033	3.3 ± 1.2	2.9 ± 1.1	0.092
Job satisfaction							
Next 5 years	3.9 ± 1.1	4.6 ± 0.7	3.8 ± 1.1	0.028	3.9 ± 1.2	3.9 ± 0.9	0.963
Next 10 years	3.5 ± 1.1	4.3 ± 0.8	3.4 ± 1.1	0.017	3.7 ± 1.2	3.4 ± 1.0	0.283

*OEM* Occupational and environmental medicine; *SD* Standard deviation

^*^By Mann-Whitney test

^**^By Student’s *t*-test

^a^Mean ± SD

The proportion of residents who thought that the outlook for OEM over the next 10 years was positive decreased as the year of residency increased, with the LYG reporting 3.6 points and the HYG reporting 3.1 points. The outlook for pay over the next 10 years was 3.2 points in the LYG and 2.7 points in the HYG. The ITG rated the outlook for job satisfaction, employment, and social position over the next 5 years more highly than the HTG, and also had a more positive outlook for job satisfaction, social position, possibilities for development, and employment over the next 10 years.

## Discussion

OEM is the medical specialty devoted to the prevention and management of occupational and environmental illness and injury, as well as the promotion of worker health and productivity. OEM specialists have the medical knowledge necessary to evaluate and treat a wide spectrum of clinical problems, including specific diseases and common injuries. Such training can only be provided through dedicated residency training programs that also offer specialized training in industrial hygiene, epidemiology, ergonomics, and toxicology [19].

In Europe, the OEM specialization was developed by universities, institutes, and government regulatory agencies for health and safety. OEM, like the majority of other medical specialties, requires a 4-year period of full-time training in most European Union countries [20]. In United States and Canada, there are 31 OEM residency programs (28 in the United States and three in Canada) [21]. These programs consist of formal course work; clinical training, including work-site practicum rotations; and, in some programs, research training and experience. Clinical experience is provided through a combination of rotations through hospital-based OEM clinics and on-site industrial health facilities [22]. In Korea, coursework for OEM residencies comprises 1 year of basic education on the principles of OEM, 1 year of clinical training, 3 months of training in toxicology, 3 months of training in an analytical laboratory, and 18 months of OEM practice. Clinical training is also included in primary care training, but the implementation of theoretical and practical OEM training depends on the training institution. The training of OEM residents in a university college of medicine or graduate school of public health mainly focuses on OEM theory and research, whereas the training of OSHRI residents primarily involves epidemiological surveys of occupational diseases. In contrast, the training of residents in hospitals, including university hospitals, mostly involves OEM practice, such as special health examinations and health management services.

According to a 2008 report of the Research Council on Improvement in OEM Training [23], vast differences between hospital and institute training exist in the practical training environment, the areas covered by instructing specialists, the themes of research projects, and the training curriculum. In this study, we found that research comprised the main responsibility of OEM residents in the ITG, while OEM practice was the main responsibility of OEM residents in the HTG. This was because the ITG primarily received training in OEM theory through papers or research reports, while the HTG mostly received on-site practical training, such as special health examinations and health management services. The higher prevalence of off-site work days for residents in the HYG was likely because under the Occupation Safety and Health Act, only 3rd-year or higher OEM residents are allowed to conduct special health examinations and only 4th-year or higher OEM residents are allowed to conduct health management services, leading to more off-site work days for 3rd-year and 4th-year residents. A survey assessing OEM residency training in United States and Canada showed that 81 % of residency graduates were satisfied with their OEM residency training (48 % were very satisfied and 33 % were somewhat satisfied) [2]. Moreover, they expressed a higher level of satisfaction with the following residency programs: professionalism; hazard recognition, evaluation, and control; epidemiology; medical knowledge; workplace health and surveillance; toxicology; and environmental health. However, they expressed a lower level of satisfaction with the following residency programs: aerospace medical knowledge, system-based practice, practice-based learning, and health services administration [2]. In previous studies dealing with OEM residency programs in Korea, Ha et al. [8] reported a lower level of satisfaction with the following training programs: environmental disease, management of the working environment, occupational toxicology, and clinical training in general surgery. Hong [23] gave a presentation arguing that residency programs in Korea have inadequacies regarding OEM practice and clinical training. As in previous studies, the respondents in this study indicated a lower level of satisfaction with the following training programs: toxicology practice (measurement of biological markers, metabolites, and workplace environments), OEM practice (environmental diseases), and clinical training in surgery.

We hypothesize that the reason for low satisfaction with toxicology practice is that OEM residents do not have the opportunity to carry out work in the field, such as conducting working environmental measurements or analyses, and do not have access to related cases. Current toxicology practice training cannot comprehensively include the content that is required of OEM residents, and vast differences among institutions exist in what is taught. This indicates that improving the quality of toxicology at authorized institutions is urgently necessary. In recent years, training in risk assessment and risk communication has been required in order to ensure that OEM specialists are able to convey the correct toxicological information about controversial chemicals. Although the clinical practice of environmental medicine is expected to be an important specialty in the future, we found that OEM residents expressed a lower level of satisfaction with the residency training program dealing with environmental diseases. In 2014, the American College of Occupational and Environmental Medicine suggested that skills in recognizing, evaluating, and treating the adverse health effects of toxic agents in the environment be included as OEM competencies [24]. The relatively low levels of satisfaction with OEM practice training (environmental diseases) were probably because it is rare for most OEM residents to diagnose and treat environmental diseases. In order to solve this problem, it is necessary to provide opportunities for residents to be involved in outpatient treatment through a consultation system with other specialties that deal with environmental diseases. The Korean Society of OEM should develop guidelines for diagnosing and managing environmental diseases. Moreover, the implementation of OEM-driven environmental health centers may be a good method for increasing satisfaction in this regard.

With regard to the clinical training of OEM residents, lower levels of satisfaction were expressed for surgical training than for medical training, which is in contrast with the dynamics observed among family medicine residents, among whom higher levels of satisfaction were expressed regarding surgical training than for medical training [15]. Most surgical training for family medicine residents was performed at secondary hospitals, leading to opportunities to train in the minor surgical methods required in primary care. In contrast, surgical training for OEM residents was performed at university hospitals and therefore did not teach the necessary skills for surgical treatment in primary care or in workplaces, leading to a low level of satisfaction with surgical training. In clinical training, close collaboration and feedback between the clinical department and the training institution is necessary. As soon as the clinical department and the hospital are designated, the required curriculum for OEM residents should be provided to the hospital so that appropriate training can be implemented. Meanwhile, individual residents should be reminded of the goal of clinical training.

In this study, we found that OEM residents preferred to work in institutions providing special health examinations or health management services, such as hospitals or KIHA/KMI, but generally did not prefer to open their own clinic or to work in public institutions (Table 3). OEM residents stated that pay and quality of life were important factors affecting their career choices. Thus, institutions providing special health examinations or health management services that meet both criteria seemed to be preferred. The high scores of hospitals and KIHA/KMI as preferred institutions also seem to be explained by these considerations. The low preference for opening one’s own clinic seems to be because the clinical practice of OEM, excluding special health examinations, fitness for work evaluations, and work-relatedness evaluations, is rare. Clinical medicine must combine both academic theory and medical practice, and OEM as a medical specialty has a particular requirement for this kind of combination for it to continue to develop as a field of clinical medicine [13]. In the United States, 51.6 % of OEM specialists work in a clinical setting, followed by corporations/industry (16.3 %), consulting (11.5 %), government (9.4 %), academia (6.6 %), and other settings (4.6 %) [25]. In order for OEM physicians to enter clinical practice, including opening their own clinics, it may be helpful for the scope of clinical practice in fields such as toxicology or environmental diseases to be further expanded. Furthermore, fitness for work evaluations and work-relatedness evaluations should be more actively conducted in clinical OEM settings.

In a study conducted by Shin et al. [26], OEM residents were found to have a positive outlook for OEM, but in this study, a positive outlook was only predominant for the next 5 years, while a negative view was predominant for the next 10 years. The outlook regarding job satisfaction over the next 10 years was positive, but the outlook for pay, social position, and employment was negative, indicating that respondents were satisfied with OEM work in and of itself, but were not satisfied with the social and financial compensation received by OEM physicians. This seems to reflect instability due to a long-term surplus in the supply of OEM physicians. Recently, the introduction of special health examinations for night shift workers has raised concerns regarding the supply of OEM physicians; nonetheless, the supply of OEM physicians is likely to exceed the demand within the next 5 years [27]. The concern of OEM residents that the employment outlook for the next 10 years will be negative appears to be related to the employment instability that will occur when the supply of OEM physicians becomes saturated. As such, the Korean Society of OEM will have to make efforts to conduct research about how to expand the target groups receiving special health examinations and the role of OEM physicians. It will also be necessary to plan systematically for the predicted supply and demand of OEM specialists in Korea.

OEM physicians are especially adept in the skills of toxicology, industrial hygiene, and epidemiology, which are directly relevant to their practice [1]. Based on reports that indicate that OEM physicians are more skilled and competitive in these domains than general practitioners [1, 28], the easing of qualifications for special health examination doctors might lead to lower-quality occupational health services. Occupational health services ensure the health of workers, and OEM physicians are the core workforce for such services. In the United States, 70 % of physicians enrolled in academic-based OEM residency programs are financially supported by National Institute of Occupational Safety and Health [29]. Following the example of the United States, we should provide financial assistance to OEM residents in light of the public interest in occupational health.

To the best of our knowledge, this is the second study evaluating the training programs of OEM residents in Korea. Our findings are noteworthy, but several potential limitations exist in this study. Only 88 of the 125 OEM residents (70.4 %) surveyed in August 2012 provided responses suitable for analysis, meaning that it is not possible to eliminate the possibility that respondents and non-respondents had significantly different perspectives. Even in the questionnaires that were used in the final analysis, the answers to some questions were absent. In categorizing training institutions into the ITG and HTG, university hospitals had characteristics similar to hospital training sites, but also had some characteristics, such as research requirements, that were similar to research institutes. Moreover, no qualitative analysis of the study subjects was performed.

Nonetheless, the results of this study are meaningful because they reflect the opinion of OEM residents themselves, which is useful for identifying directions for future improvement. The results of this study may help guide future discussion on alternative or additional OEM training needs. Research on the instructors at OEM training institutions will also be needed to understand resident training, although this study did not address that issue. Doing so is particularly important because the passion and commitment of professors and instructing specialists play a critical role in the training of residents, and when the workload of instructors is too heavy, resident training cannot be the top priority.

## Conclusion

This study found significant differences in the training environment and satisfaction of OEM residents between the ITG and HTG. The actual training content for OEM residents must be standardized, and the quality of training must be improved by holding a regional conference and introducing open training methods, such as an open hospital system. The Korean Society of OEM will need to provide practical and systemic training on special health examinations and health management services. The Korean-language OEM textbook may also play a role in reducing educational disparities among training institutions. OEM residency programs must be improved, with a focus on toxicology practice, OEM practice (especially environmental diseases), and clinical training (especially surgical training). In addition, the development of outpatient clinical practices in the areas of toxicology or environmental diseases may be helpful for establishing the identity of OEM as a discipline. The findings of this study shed light on areas that require improvement in OEM residency programs and provide a valuable perspective on the strengths and weaknesses of each type of training institution.

## Abbreviations

OEM: Occupational and environmental medicine
ITG: Institute training group
HTG: Hospital training group
LYG: Lower-year group
HYG: Higher-year group
KIHA: Korean Industrial Health Association
KMI: Korea Medical institute
OSHRI: Occupational Safety and Health Research Institute
